# Radiosensitivity Prediction of Tumor Patient Based on Deep Fusion of Pathological Images and Genomics

**DOI:** 10.3390/bioengineering13020142

**Published:** 2026-01-27

**Authors:** Xuecheng Wu, Ruifen Cao, Zhiyong Tan, Pijing Wei, Yansen Su, Chunhou Zheng

**Affiliations:** 1 Key Laboratory of Intelligent Computing and Signal Processing, Ministry of Education, School of Computer Science and Technology, Anhui University, Hefei 230601, China; e23301235@stu.ahu.edu.cn (X.W.); e24101005@stu.ahu.edu.cn (Z.T.); 2 State Key Laboratory of Pathogenesis, Prevention and Treatment of High Incidence Diseases in Central Asia, Xinjiang Medical University, Urumqi 830054, China; suyansen@ahu.edu.cn (Y.S.); zhengch99@126.com (C.Z.); 3 Physical Science and Information Technology, Anhui University, Hefei 230601, China; weipj@ahu.edu.cn; 4 School of Artificial Intelligence, Anhui University, Hefei 230601, China

**Keywords:** radiosensitivity, histopathological images, genomic feature, multimodal fusion

## Abstract

The radiosensitivity of cancer patients determines the efficacy of radiotherapy, and patients with low radiosensitivity cannot benefit from radiotherapy. Therefore, accurately predicting radiosensitivity before treatment is essential for personalized and precise radiotherapy. However, most existing studies rely solely on genomic and clinical features, neglecting the tumor microenvironmental information embedded in histopathological images, which limits prediction accuracy. To address this issue, we propose Resfusion, a deep multimodal fusion framework that integrates patient-level gene expression profiles, clinical records, and histopathological images for tumor radiosensitivity prediction. Specifically, the pre-trained large-scale pathology model is used as an image encoder to extract global representations from whole-slide pathological image. Radiosensitivity-related genes are selected using an autoencoder combined with univariate Cox regression, while clinically relevant variables are manually curated. The three modalities are first concatenated and then refined through a self-attention-based module, which captures inter-feature dependencies within the fused representation and highlights complementary information across modalities. The model was evaluated using five-fold cross-validation on two common tumor datasets suitable for radiotherapy: the Breast Invasive Carcinoma (BRCA) dataset (282 patients in total, with each fold partitioned into 226 training samples and 56 validation samples) and the Head and Neck Squamous Cell Carcinoma (HNSC) dataset (200 patients in total, with each fold partitioned into 161 training samples and 39 validation samples). The average AUC values obtained from the five-fold cross-validation reached 76.83% and 79.49%, respectively. Experimental results demonstrate that the Resfusion model significantly outperforms unimodal methods and existing multimodal fusion methods, verifying its effectiveness in predicting the radiosensitivity of tumor patients.

## 1. Introduction

Malignant tumors, also known as cancers, are among the leading causes of death worldwide, accounting for approximately one-sixth of global deaths [1,2,3]. As a main treatment mean, radiotherapy is administered to nearly 70% of cancer patients during the course of their disease [4,5]. However, in clinical practice, owing to inter-individual biological heterogeneity, the therapeutic efficacy of radiotherapy varies significantly among patients [6]. Radiosensitive patients can achieve tumor control via radiotherapy, whereas radioresistant patients not only exhibit poor treatment outcomes but may also suffer from severe complications induced by radiation [7,8]. For instance, approximately 15% of patients with HNSCC experience local recurrence due to radio resistance, and around 10% of BRCA patients receiving radiotherapy develop breast tissue damage of varying severity [9,10,11]. Such inter-individual variability poses enormous challenges to clinical decision-making: if clinicians cannot accurately determine whether patients will benefit from radiotherapy prior to treatment, some patients will be exposed to the risks of ineffective therapy. Therefore, accurate pre-treatment prediction of tumor radiosensitivity in cancer patients is of great importance.

In recent years, increasing attention has been paid to predicting tumor radiosensitivity, as understanding molecular determinants of radiotherapy response is crucial for individualized treatment. Some studies have shown that miRNAs and some target genes are related to the radiotherapy results of patients. For instance, Ma et al. have revealed the methylation characteristics of four genes related to radiotherapy, which can be used to predict the survival of patients with HNSCC, providing potential therapeutic targets for new treatment methods for HNSCC [12,13]. Liu et al. combined multiple omics data of 122 differential genes with clinical outcomes to establish a 12 radiosensitivity genes signature by two-stage regularization and multivariable Cox regression models [14]. Chen et al. used univariate Cox regression analysis and lasso Cox regression method to screen optimal gene for constructing a radiosensitivity estimation signature, and combined with independent prognostic factors to predict the 1-, 3-, and 5-year OS of radiation-treated BRCA patients [15]. Li et al. selected glmboost + naivebayes model to build the radiosensitivity score based on 18 key genes through the evaluation of 113 machine learning algorithm combinations, which demonstrated good predictive performance in both public and in-house datasets [16]. Although these studies have advanced the understanding of radiosensitivity from a genomic perspective, they primarily focus on molecular features while neglecting the impact of the tumor microenvironment and morphological heterogeneity. Consequently, the predictive performance of existing models remains limited, underscoring the need for multimodal approaches that integrate histopathological and molecular information.

Histopathological images capture detailed information on cellular morphology, spatial organization, and the tumor microenvironment, all of which play a critical role in determining treatment response. In recent years, substantial progress has been made in extracting informative features from whole-slide images (WSIs) for cancer diagnosis and prognosis. For instance, Xu et al. constructed an image feature extractor using the DINOv2-LongNet network, which performed well in survival analysis tasks [17]. Song et al. compressed thousands of patches from a WSIs based on Gaussian mixture models and achieved good results in cancer subtype classification and survival prediction [18]. Furthermore, Chen et al. and Yang et al. introduced self-distillation and masked image modeling strategies, respectively, enhancing the generalization capability of pathology feature extractors [19,20,21]. These advances demonstrate that deep pathology models can effectively characterize tumor microenvironmental heterogeneity, providing a promising foundation for integrating histopathological information into radiosensitivity prediction frameworks. Nevertheless, the integration of image-derived microenvironmental representations into radiosensitivity modeling remains largely unexplored, leaving significant potential for improvement.

With the rapid advancement of deep learning, multimodal fusion has emerged as a powerful paradigm for cancer prognosis and treatment-response modeling. Several studies have demonstrated that integrating heterogeneous data sources—such as histopathology, genomics, radiomics, and clinical information—can substantially enhance predictive performance. For example, Nicolas et al. established a prediction model for non-small cell lung cancer immunotherapy outcomes by integrating clinical, pathological, radiological, and transcriptomic data, achieving good performance [22]. Song et al. summarized the morphological content of WSIs by condensing its constituting tokens using morphological prototypes, and processed the multimodal tokens obtained by the fusion network, achieving excellent performance in survival analysis tasks [23]. Chen et al. used CNN to extract pathological image features, GCN to extract cell map features, processed genomic data through self-normalization network, and established a survival analysis model for renal cell carcinoma using Kronecker product fusion [24]. Despite these advances, studies specifically addressing radiosensitivity prediction through multimodal integration remain limited. For example, Dong et al. proposed a model combining pathological and genomic features but fused only risk scores from each modality, failing to capture deeper cross-modal interactions [25]. Similarly, Yu et al. used random forest stacking to fuse 10 related genes and 8190 pathological features, and established a model for predicting the radiosensitivity of non-small cell lung cancer patients, yet the fusion remained shallow and heuristic [26]. These limitations underscore the need for a unified deep fusion framework capable of jointly learning complementary information from histopathological, genomic, and clinical modalities to improve radiosensitivity prediction accuracy.

In recent years, research on radiosensitivity prediction has focused primarily on unimodal genomic features, ignoring the value of pathological images. Several multimodal studies have attempted to integrate pathological and genomic data, but their fusion methods are limited to risk score stacking or shallow feature concatenation, failing to capture the deep inter-modal correlations and thus resulting in limited prediction accuracy. Although existing multimodal deep fusion models achieve comprehensive data integration, they lack targeted optimization for radiosensitivity prediction and cannot meet the requirements of clinical applications.

To address the limitations of existing studies on tumor radiosensitivity prediction, we propose a deep learning-based multimodal fusion framework that integrates histopathological images, gene expression, and clinical information to achieve accurate and individualized prediction of radiosensitivity. Specifically, the slide-level representations from whole-slide images are extracted using the Prov-GigaPath large-scale pathology foundation model. Key gene features associated with radiosensitivity are identified and extracted through an autoencoder combined with univariate Cox analysis. These heterogeneous features are subsequently fused via a self-attention-based architecture that adaptively reinforces complementary inter-modal relationships while suppressing redundant information, thereby enhancing the overall predictive performance. The proposed method is validated on BRCA and HNSC datasets, representing two major anatomical sites the breast and head-neck regions demonstrating its effectiveness and potential for clinical application in personalized radiotherapy.

## 2. Method

### 2.1. Model Framework

**Figure 1:**
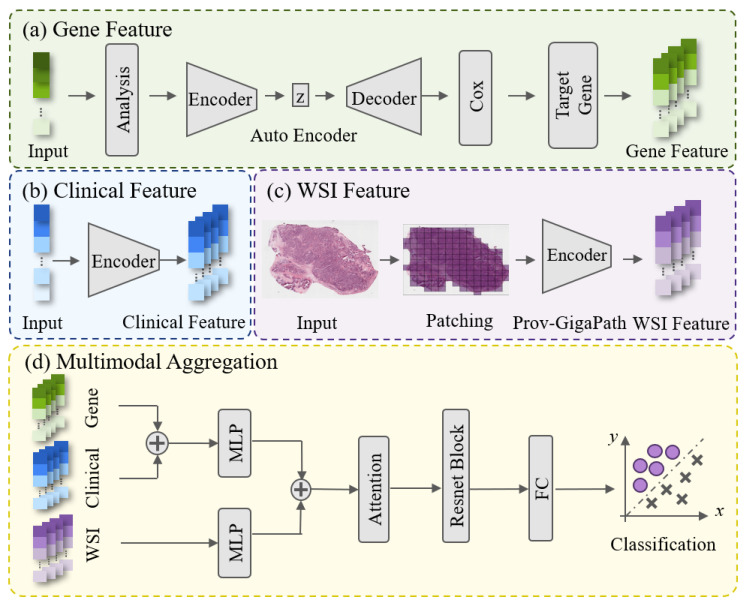
Overview of the Resfusion workflow.

An overview of the proposed multimodal framework for predicting tumor radiosensitivity is illustrated in Figure 1. The framework integrates histopathological, genomic, and clinical information to comprehensively characterize tumor heterogeneity and treatment response potential.

To effectively capture tumor microenvironmental and morphological characteristics, the large-scale foundation model Prov-GigaPath is employed to extract slide-level representations from whole-slide images [27,28]. In the genomic branch, radiosensitivity-related genes are identified through a three-stage feature selection strategy combining differential expression analysis, autoencoder-based dimensionality reduction, and univariate Cox regression analysis. In the clinical branch, variables associated with tumor progression, such as patient age, sex, and tumor stage are manually selected and numerically encoded. Finally, a self-attention-based fusion module is designed to integrate the extracted features from the three modalities. This architecture adaptively emphasizes complementary cross-modal information and suppresses redundant signals, resulting in a more robust and accurate prediction of tumor radiosensitivity.

### 2.2. Pathological Image Feature Extraction

Whole-slide images (WSIs) are extremely large and cannot be directly processed by deep learning models due to their high resolution and gigapixel scale. To efficiently extract morphological and microenvironmental information, we employed the Prov-GigaPath foundation model [17], a large-scale whole-slide pathology encoder pre-trained on more than 1.3 billion image tiles from diverse cancer types. Benefiting from its extensive pretraining and large-scale cross-cancer coverage, Prov-GigaPath is capable of generating robust and generalizable representations that capture both fine-grained cellular morphology and global tissue context. This enables downstream models to leverage comprehensive pathological information without requiring task-specific retraining [27,28]. For each WSI, the pre-trained model directly generates a slide-level feature embedding that captures comprehensive spatial and contextual representations as illustrated in Figure 1c. The resulting 768-dimensional embedding P was used as the pathological modality input for subsequent multimodal fusion and radiosensitivity prediction. The mathematical formulation of the slide-level feature extraction process is given as follows:

(1) $$P=F_{P}\left(T\left(w\right)\right),P\in R^{768}$$

where w denotes an individual whole-slide image (WSI), T represents the tile partitioning operation that segments a WSI into standardized image patches, $F_{P}$ refers to the feature encoding function of the pre-trained Prov-GigaPath model, and P denotes the 768-dimensional slide-level pathological feature embedding.

### 2.3. Genomic Feature Extraction

In this study, a three-stage pipeline combining differential expression analysis, autoencoder-based dimensionality reduction, and Cox regression analysis was employed to identify radiosensitivity-related genes, as illustrated in Figure 1a. First, differential expression analysis was conducted to identify genes exhibiting significant expression changes before and after radiotherapy, as these genes are likely to be associated with radiation response. Second, the selected differentially expressed genes were input into an autoencoder network to extract compact and representative features while minimizing noise. The encoder consisted of two fully connected layers (1024 and 512 neurons), followed by a 100-dimensional bottleneck layer that compresses high-dimensional gene data into key latent representations. The decoder was architecturally symmetric to the encoder, enabling the reconstruction of the original input and facilitating the learning of meaningful latent representations via the minimization of reconstruction loss. Finally, a univariate Cox proportional hazards regression was applied to the latent features generated by the autoencoder to evaluate their association with patient survival outcomes. This step identifies genes whose expression levels are significantly correlated with overall survival, indicating their potential relevance to tumor radiosensitivity. The subset of genes passing the Cox significance threshold was defined as radiosensitivity-associated genes, and their expression profiles were used as the genomic feature vectors for multimodal fusion. The entire process of radiosensitivity-related gene identification can be succinctly formulated as:

(2) $$G=\mathrm{Cox}\left(\mathrm{Auto}(z)\right),\;G\in R^{9}$$

where z denotes the initial set of gene expression profiles before screening. Auto(.) represents the autoencoder-based dimensionality reduction and feature extraction operation, Cox(.) refers to the Cox proportional hazards regression for survival correlation screening, and G denotes the final 9-dimensional radiosensitivity-associated gene feature vector.

### 2.4. Selection of Clinical Features

In clinical practice, patient-specific variables—such as age, gender, tumor stage, and pathological characteristics—have a critical influence on radiotherapy planning and treatment outcomes [29]. Based on their clinical relevance, these parameters were selected as candidate features for modeling tumor radiosensitivity, as illustrated in Figure 1b.

Prior to feature encoding, data cleaning and selection were performed to ensure reliability and interpretability. Clinical variables with excessive missing values were excluded to maintain data completeness, while features with low variance or limited discriminatory power were removed to reduce redundancy. The final set of clinical variables was determined according to feature availability and clinical relevance in each dataset. For the HNSC cohort, the selected variables comprised age, clinical stage, tumor grade, pathological T stage, and pathological N stage. For the BRCA cohort, the retained variables included age, pathological grade, and pathological M, N, and T stages. These variables represent clinically meaningful indicators of tumor progression and patient condition. Subsequently, the selected features were input into a fully connected encoder network, which projects the clinical attributes into a latent feature space. The resulting encoded clinical representations serve as the clinical modality input for multimodal fusion. The clinical feature encoding process can be succinctly formulated as follows:

(3) $$C=\mathrm{FC}\left(S(v)\right),\;C\in R^{6}$$

where v denotes the raw set of patient-specific clinical variables, S(.) represents the data cleaning and feature selection procedure, FC(.) refers to the fully connected encoder network, and C is the 6-dimensional encoded clinical feature vector for multimodal fusion.

### 2.5. Multimodal Features Fusion

**Figure 2:**
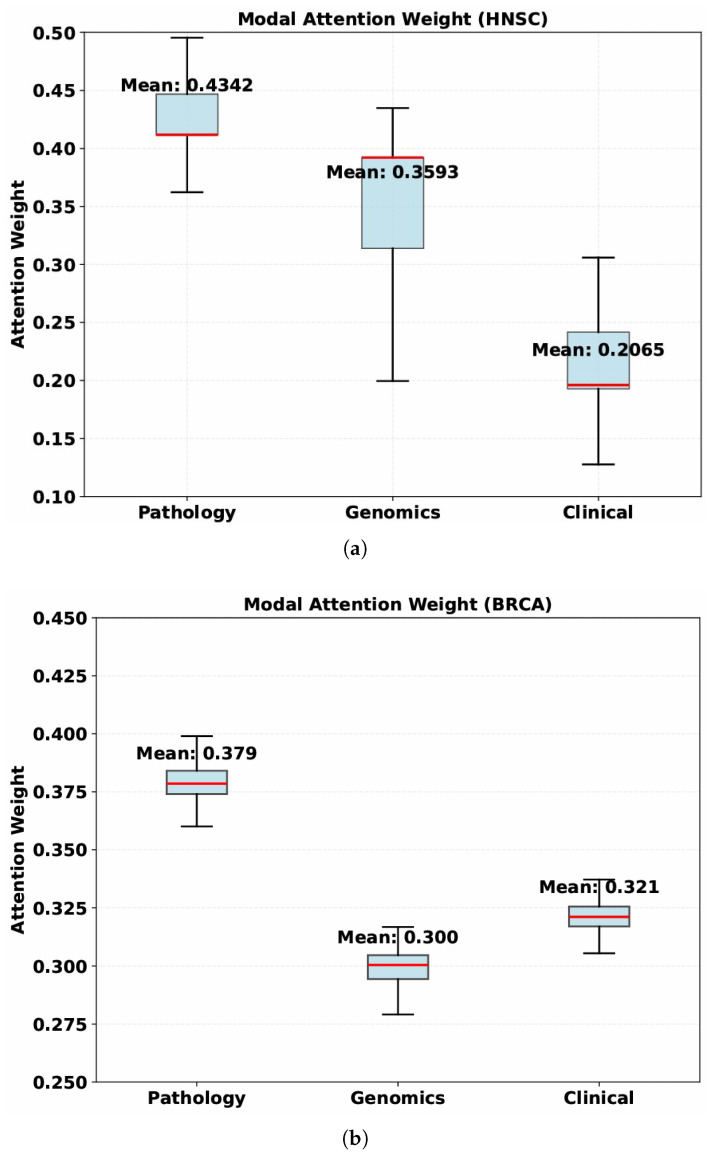
(**a**) Modal attention weight distribution on the HNSC dataset. (**b**) Modal attention weight distribution on the BRCA dataset.

To achieve effective integration of heterogeneous features and mitigate the imbalance caused by differences in feature dimensionality, a hierarchical multimodal fusion strategy was adopted, as illustrated in Figure 1d. First, genomic features and clinical features, which share relatively close dimensional scales and biological relevance, are concatenated and passed through a multi-layer perceptron (MLP) to generate an intermediate fused repre sentation. Second, this joint representation is further integrated with the WSIs features extracted by the Prov-GigaPath encoder. An additional MLP layer is employed to project the high-dimensional WSIs features into the same latent space as the fused genomic-clinical representation, ensuring dimensional consistency for subsequent multimodal interaction. Subsequently, a self-attention module is applied to the concatenated multimodal represen tation to capture inter-feature dependencies and emphasize informative patterns within the fused feature space.This module can adaptively assign distinct weights to different modalities, a property that is further verified by the weight distribution results of the aforementioned two datasets—specifically, the attention weight distribution on the Head-Neck Squamous Cell Carcinoma (HNSC) dataset is presented in Figure 2a, and that on the Breast Invasive Carcinoma (BRCA) dataset in Figure 2b. A learnable weight layer further refines the aggregated representation before the final classification. The overall fusion process can be formulated as follows:

(4) $$\begin{array}{c} F_{\mathrm{concat}}=\left[\mathrm{MLP}\left(\left[G;C\right]\right)\;;\;\mathrm{MLP}\left(P\right)\right] \end{array}$$

(5) $$\begin{array}{c} F_{\mathrm{att}}=\mathrm{SelfAttn}\left(F_{\mathrm{concat}}\right) \end{array}$$

(6) $$\begin{array}{c} F_{\mathrm{weighted}}=W⊙F_{\mathrm{att}} \end{array}$$

(7) $$\begin{array}{c} \hat{y}=\mathrm{Softmax}\left(\mathrm{MLP}\left(F_{\mathrm{weighted}}\right)\right) \end{array}$$

where G, C and P denote the genomic, clinical, and pathological representations, respectively; SelfAttn(.) captures internal dependencies within the fused representation; W represents learnable weights used for adaptive feature reweighting; ⊙ represents element-wise weighting; and $\hat{y}$ is the final prediction of tumor radiosensitivity.

## 3. Result

To verify the performance of proposed method, HNSC and BRCA datasets were used to train and test the model. Given the limited sample size and class imbalance in both datasets, five-fold cross-validation was performed to ensure robustness and reduce the impact of data partition bias. Specifically, the samples were randomly divided into five subsets, with four folds used for training and one for testing in each iteration. The average performance across the five folds of cross-validation were shown in this study. The dataset, experimental parameters, results of comparative experiments and ablation experiments are described in detail below.

### 3.1. Data Collection and Preprocessing

All the histopathological image data, gene expression data and clinical report data of cancer patients after radiotherapy were downloaded from TCGA database [30]. In order to avoid the impact of unrelated causes of death, we removed the samples with a survival time of less than 30 days, and finally screened 200 patients with HNSCC and 282 patients with BRCA who received radiotherapy.

How to define whether a patient is sensitive to radiotherapy is a key point for this study. According to studies and clinical practice [2,3,4], the radiosensitivity of patients was defined and classified according to their survival outcomes following radiotherapy. The patients who survived for more than five years after radiotherapy were defined as patients sensitive to radiotherapy and were used as positive samples. The patients who died within five years after radiotherapy were regarded as negative samples. It should be noted that this binary definition is a pragmatic surrogate based on clinical survival outcomes, as it does not distinguish between tumor-related and non-tumor-related causes of death, nor does it exclude the potential impact of combination therapies (e.g., surgery, chemotherapy, immunotherapy) administered alongside radiotherapy. Therefore, the ’radiosensitivity’ predicted in this study reflects the clinical outcome after radiotherapy rather than pure biological radiosensitivity of the tumor itself, which is a simplification of the complex biological phenomenon for prognostic modeling purposes. Finally, the head and neck cancer dataset contains a total of 200 patients who received radiotherapy, and there are 149 positive samples and 51 negative samples for training and testing. The breast cancer dataset consists of 282 cases, in which the positive and negative samples respectively are 239 and 43 cases. The details of dataset as shown in Table 1.

### 3.2. Imbalanced Data Handling

We found that there were more cases with survival times exceeding five years and fewer with shorter survival times in the used datasets, indicating a severe imbalance in the ratio of positive to negative samples. This imbalance would pose a challenge to the training of machine learning models. To solve this problem, during the training process, the contribution of each sample to the loss function is calculated by taking the reciprocal of the proportion of such samples in the total number. This approach assigns higher loss weights to the minority samples, enabling the model to pay more attention to the difficult-to-classify negative samples during training and improving the recognition ability for the minority samples.

The loss function with weight can be expressed as:

(8) $$\mathrm{WeightedLoss}\left(p,t,w\right)=-\frac{1}{N}\sum_{i=1}^{N}w_{i}\left[t_{i}\log\left(p_{i}\right)+\left(1-t_{i}\right)\log\left(1-p_{i}\right)\right]$$

where N is the number of samples; $p_{i}$ is the probability that the model predicts a positive class for the sample, $t_{i}$ is the real label of the sample, and $w_{i}$ is the weight coefficient.

### 3.3. Evaluating Criteria

The metrics including Recall, Precision, F1 and Accuracy are used to evaluate the model. Their calculation formula is as follows:

(9) $$\mathrm{Precision}=\frac{\mathrm{TP}}{\mathrm{TP}+\mathrm{FP}}$$

(10) $$\mathrm{Recall}=\frac{\mathrm{TP}}{\mathrm{TP}+\mathrm{FN}}$$

(11) $$F1=\frac{2\mathrm{TP}}{2\mathrm{TP}+\mathrm{FP}+\mathrm{FN}}$$

(12) $$\mathrm{Accuracy}=\frac{\mathrm{TP}+\mathrm{TN}}{\mathrm{TP}+\mathrm{TN}+\mathrm{FP}+\mathrm{FN}}$$

TP (true positive) represents the correctly predicted number of patients sensitive to radiotherapy; TN (true negative) represents the correctly predicted number of patients insensitive to radiotherapy; FP (false positive) represents the number of patients who were incorrectly predicted to be sensitive to radiotherapy; FN (false negative) represents the number of patients who are not sensitive to radiotherapy, which is incorrectly predicted. In addition, this study also uses AUC (the area under the receiver operating characteristic (ROC) curve) to evaluate the overall performance of the model.

Generally, Recall, Precision, F1 and Accuracy are affected by thresholds. When the probability value of the model output is greater than or equal to this value, it is predicted as a positive sample, otherwise it is predicted as a negative sample. In the study, the threshold was set to 0.5.

### 3.4. Implementation Details

The proposed method was implemented using PyTorch 2.4.1. The model was optimized using the Adam optimizer with a learning rate of 0.001 and trained for 700 epochs under the cross-entropy loss function. All experiments were conducted on a single NVIDIA GeForce RTX 4090 GPU.

### 3.5. Predictive Performance Comparison

The Resfusion model is proposed to predict the radiosensitivity of cancer patients by integrating histopathology, gene expression, and clinical variables. To evaluate the performance of Resfusion, two recent multimodal survival models including MMP and Dyam were selected as comparative baselines. The MMP model fused gene-expression profiles with histopathological images to forecast cancer patient prognosis. Dyam further enriched this paradigm by integrating genomic, pathological, and clinical data into a unified prognostic framework.

**Figure 3:**
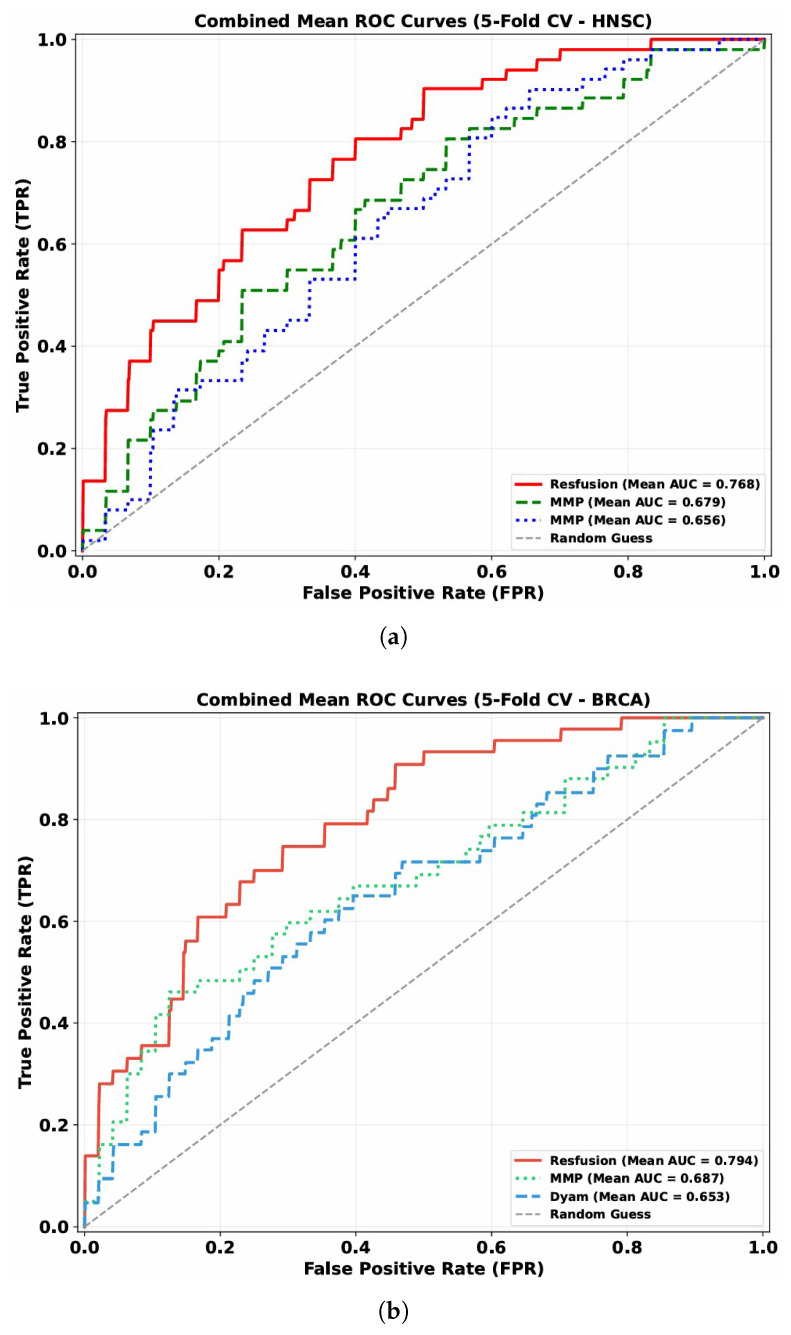
(**a**) Mean ROC curve of 5-fold cross-validation on the HNSC dataset. (**b**) Mean ROC curve of 5-fold cross-validation on the BRCA dataset.

The discriminative capability of these models is visually illustrated by the mean ROC curves (5-fold cross-validation) across the HNSC and BRCA datasets (Figure 3): Resfusion consistently exhibits a more favorable curve position compared to MMP and Dyam, aligning with the quantitative performance metrics.

Table 2 and Table 3 present the performance of MMP, Dyam and Resfusion models. Compared with the MMP and Dyam models on the HNSC dataset, the model improved Precision by about 7.07% and 12.51%, Recall by about 9.22% and 13.09%, AUC by about 8.88% and 11.15%, Accuracy by about 3.87% and 9.47%, and F1-score by about 7.74% and 12.43%, respectively. On the BRCA dataset, the model improved Precision by approximately 14.32% and 19.35%, Recall by approximately 21.00% and 25.12%, AUC by approximately 10.75% and 14.18%, Accuracy by approximately 12.80% and 20.00%, and F1-score by approximately 17.43% and 22.01%, respectively. These results confirm the superior generalization and predictive capability of the proposed multimodal framework across different cancer datasets, demonstrating its robustness and potential clinical applicability in tumor radiosensitivity prediction.

### 3.6. Performance Comparison of Pathology Feature Extractors

This section focuses on the influence of different pathological image feature extraction methods on radiosensitivity prediction performance. For this purpose, we employed three published and well-trained self-supervised learning algorithms, Prov-GigaPath, UNI, and Panther, as feature extractors for pathological images. Specifically, UNI applies attention-based aggregation to obtain slide-level representations, whereas Panther leverages Gaussian mixture modeling to cluster image patches and generate slide-level features. To evaluate these extracted features, we trained our proposed Resfusion model on them and compared their performance in predicting tumor radiosensitivity.

As shown in Figure 3, the model showed the best performance when using the features extracted by Prov-GigaPath model. In the 5-fold cross validation, the model achieved 76.83% AUC on the HNSC dataset and 79.49% AUC on the BRCA dataset, which showed the robustness of the features extracted by Prov-GigaPath model in the analysis of tumor radiosensitivity. Accordingly, the histopathological features extracted using the Prov-GigaPath foundation model were employed as the image modality input of the Resfusion framework.

### 3.7. Ablation Experiment

To evaluate the contribution of each data modality to the overall performance of Resfusion, we conducted a series of ablation experiments. Based on the full multimodal model, features from each modality—genomic (G), histopathological image (I), and clinical report (R)—were selectively removed to assess their individual impact. Specifically, five-fold cross-validation was performed for each ablation configuration, and model performance was compared on the test sets. Image (I) means using only image features, gene (G) means using only gene features, and report (R) means using only clinical report features, Image + Gene (I + G) means that the clinical features are removed, only image and gene features are used for model training and evaluation. Similarly, Image + Report (I + R) means that the genetic features are removed on the basis of this model, only image and report features are used for model training and evaluation. Gene + Report (G + R) means that image features are removed, only gene and report features are used for model training and evaluation. Image + Gene + Report (I + G + R) means using all features.

It can be seen from the results in Table 4 that in HNSC, when the model only uses gene and clinical report features, the AUC decreased by 10.32%; when the model only uses image and clinical report features, the AUC decreases by 9.21%, and when the model only uses image and gene features, the AUC decreases by 5%. Similarly, the findings in Table 5 reveal that in BRCA, the AUC dropped by 10.92% when the model only uses gene and report features, by 7.84% when using only image and report features, and by 6.44% when restricted to image and gene features.

In conclusion, the above results show that in the multimodal fusion of this model, the absence of any one modality will lead to a deterioration in the model’s performance, especially the pathological image features play a more significant role.

### 3.8. KM Result Analysis

**Figure 4:**
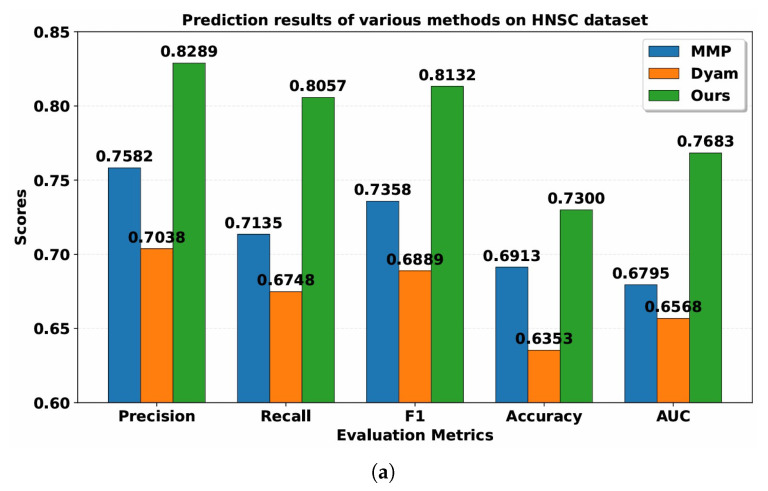

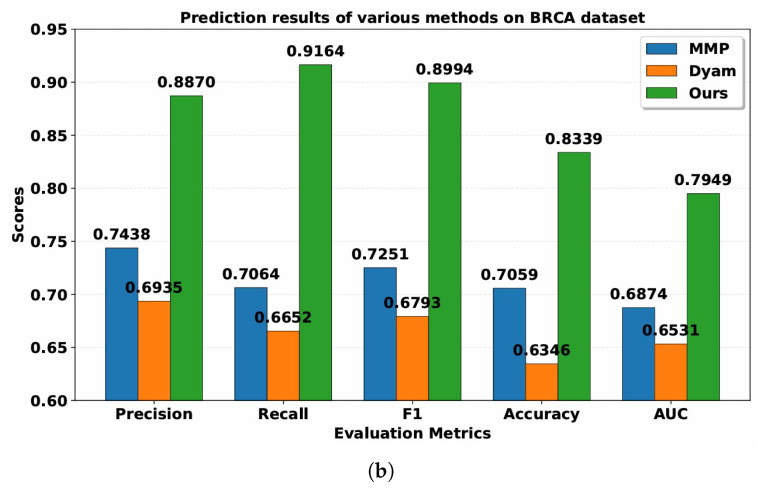
(**a**) Results of various image feature extractors on HNSC dataset. (**b**) Results of various image feature extractors on BRCA dataset.

**Figure 5:**
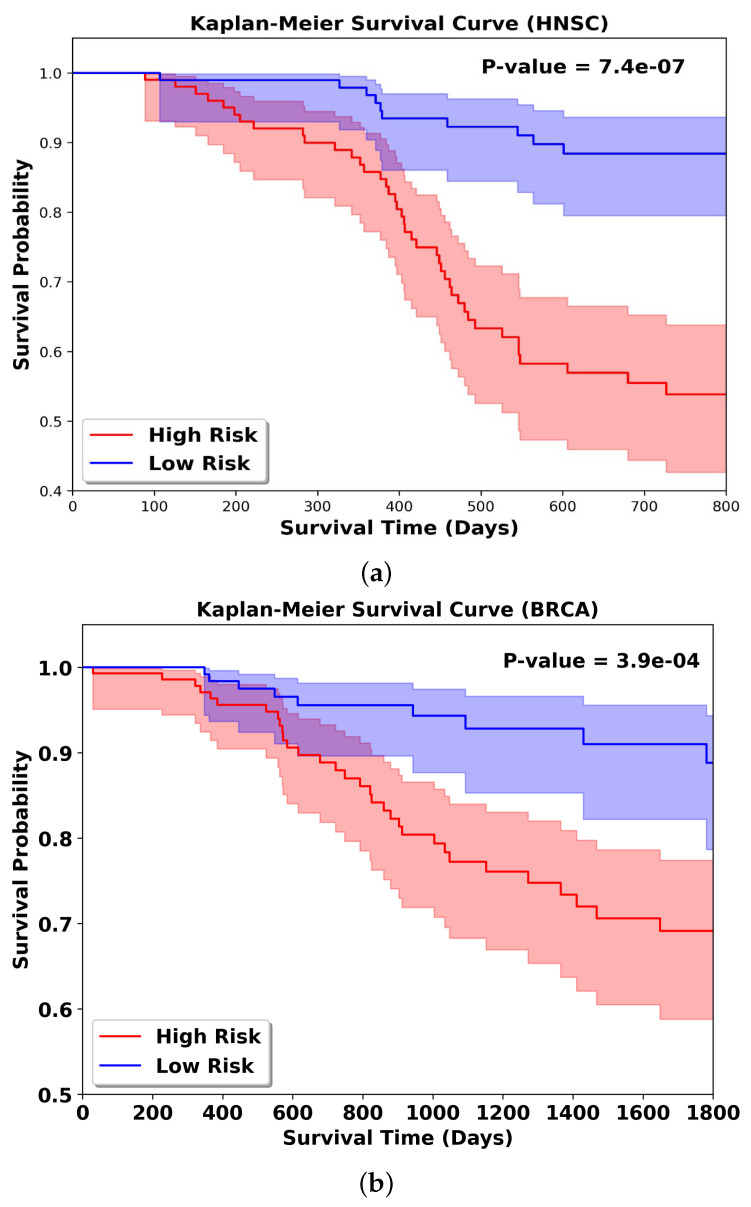
Kaplan–Meier survival curves of combined 5-fold cross-validation test sets ((**a**) HNSC dataset; (**b**) BRCA dataset).

To further validate the effectiveness of the multimodal fusion strategy in the Resfusion model, we extended the framework to a survival prediction task for radiotherapy patients, as illustrated in Figure 4. In this setting, the downstream classifier in Resfusion was replaced with a survival analysis head to estimate each patient’s risk score. Experiments were conducted using five-fold cross-validation on both the HNSC and BRCA datasets to ensure robustness. For evaluation, patients were stratified into high-risk and low-risk groups according to the median predicted risk score, and the survival differences between the two groups were assessed using standard survival metrics (e.g., log-rank test). Kaplan–Meier survival analysis was performed on two groups of patients who received radiotherapy, and the corresponding log rank test *p*-value was calculated. In the survival analysis curve, the greater the difference in survival rates between the high-risk group and the low-risk group, the better the prediction performance of the model. As shown in Figure 5, when performing radiosensitivity survival analysis using the multimodal feature fusion strategy of the Resfusion model, the model can significantly distinguish the survival differences between high-risk and low-risk patient groups: for the HNSC dataset, the log-rank test *p*-value reaches 7.4 × 10^−7^; for the BRCA dataset, the *p*-value is 3.9 × 10^−4^. This result once again proves that the Resfusion model can effectively integrate pathological image data, gene expression data and clinical reports.

## 4. Discussion

### 4.1. Comparison with Related Literature

The accurate prediction of tumor radiosensitivity is crucial for optimizing personalized radiotherapy strategies, and existing studies have explored various approaches based on unimodal or multimodal data. The Resfusion model proposed in this study integrates histopathological images, genomic features, and clinical data via a deep self-attention fusion framework, achieving superior performance compared with previous studies and providing new insights for the advancement of this field.

In terms of unimodal genomic studies, Liu et al. constructed a 12-gene radiosensitivity signature using multi-omics data and Cox regression [14]. However, this study relied solely on molecular features while neglecting the impact of the tumor microenvironment, which limited its predictive capability. Chen et al. developed a six-gene signature for breast cancer radiosensitivity, which achieved an AUC of 0.687 on the BRCA dataset [15]. In contrast, the Resfusion model in this study reached an AUC of 0.79 on the dataset of the same cancer type, fully demonstrating the significant value of integrating pathological and clinical information.

In the realm of multimodal studies, Dong et al. proposed a model for predicting breast cancer radiosensitivity by fusing pathological images and genetic data [25]. Nevertheless, the fusion strategy of this model only stayed at the level of integrating risk scores from individual modalities. This “result-level fusion” failed to explore the intrinsic correlations between pathological images and genetic data. The AUC of this model was only approximately 0.65, which was significantly lower than that of Resfusion on the same cancer type, highlighting the crucial necessity of deep cross-modal fusion.

Beyond outperforming shallow fusion methods, Resfusion also exhibits superior performance in comparison with deep fusion-based multimodal models. The MMP model predicts cancer patient prognosis by deeply fusing genetic data and pathological images, while the Dyam model conducts survival analysis through the deep integration of genetic, pathological, and clinical data. Although both models realize comprehensive data integration, they lack targeted optimization for radiosensitivity prediction. In contrast, Resfusion achieves significant improvements in all metrics of the survival analysis task compared with the MMP and Dyam models by precisely screening radiosensitivity-related genes and integrating key clinical variables closely associated with radiotherapy outcomes.

Regarding pathological image feature extraction, a comparative experiment was conducted in this study among three feature extractors: Prov-GigaPath, UNI, and Panther. The results showed that Prov-GigaPath outperformed the other two extractors significantly, achieving 5–8% higher AUC on both datasets. This indicates that Prov-GigaPath is a well-suited pathological image feature extractor for this study.

### 4.2. Limitations of the Research

Although this study has achieved promising results, it still has certain limitations. For instance, differences in the digitization pipelines of pathological images across various institutions may affect morphological features, thereby compromising the quality of image features extracted by Prov-GigaPath. Meanwhile, discrepancies in gene sequencing methods and missing values in clinical variables can also exert an impact on model accuracy. Second, defining radiosensitivity based on 5-year survival is a pragmatic yet imperfect surrogate: patients who died within 5 years may have succumbed to non-tumor-related causes, while some other patients may experience late recurrence after the 5-year follow-up cutoff. All these factors can induce biases in the final predictive performance of the model [31,32,33,34,35].

Furthermore, the genetic features extracted in this study focus solely on gene expression levels, while neglecting fine-grained molecular data such as immunohistochemistry (IHC) markers and metabolomic profiles, which are closely correlated with radiosensitivity [31,32,33]. In addition, the dataset only includes two cancer types from a single data source, which may restrict the model’s generalizability to other cancer types. Finally, the model does not incorporate radiomic features. Radiomics can capture the anatomical and functional characteristics of tumors, which complement pathological and genomic data and are crucial for radiotherapy planning; the absence of such features limits the predictive performance to a certain extent.

### 4.3. Future Directions

To address the aforementioned limitations, this study proposes the following future research directions. First, expand the multimodal framework to integrate radiomic features derived from magnetic resonance imaging (MRI), as well as data from other modalities including immunohistochemistry (IHC) markers, metabolomics and epigenomics [31,32,33]. This will enable a more comprehensive characterization of tumor biological features and treatment responses, thereby further improving prediction accuracy. Second, collaborate with multiple clinical institutions to collect diverse datasets covering various cancer types, break through the limitation of the current single data source, and enhance the generalizability of the model.

Meanwhile, refine the definition of radiosensitivity by incorporating multiple clinical endpoints such as tumor regression rate, progression-free survival and radiation-induced toxicity, so as to establish a more comprehensive characterization system and reduce label noise caused by sole reliance on 5-year survival [35].

Finally, conduct prospective clinical trials to validate the performance of the Resfusion model in real-world clinical settings, and evaluate its practical utility in guiding radiotherapy decision-making and improving clinical outcomes.

### 4.4. Clinical Application Scenarios and Practical Value

The ResFusion model proposed in this study holds clear practical value, with its core application potential reflected in two key aspects: potential integration into clinical decision support and facilitation of medical resource optimization. Designed to predict tumor radiosensitivity using routinely available imaging, gene expression, and clinical data, the model provides individualized radiosensitivity assessments prior to radiotherapy, assisting clinicians in identifying patients who are more likely to respond favorably or unfavorably to radiotherapy. Such predictions can be considered as supportive information, together with established clinical factors, to inform personalized treatment planning. Importantly, the model is intended to support rather than replace clinical decision-making, and further prospective validation is required before its formal clinical application. Meanwhile, by accurately identifying patients who will truly benefit from radiotherapy, the model helps reduce unnecessary radiotherapy cases, which not only lowers medical costs associated with radiotherapy equipment occupancy and drug consumption but also addresses the critical challenge of limited medical resources—particularly in resource-constrained regions where the efficient utilization of radiotherapy facilities is paramount. Additionally, the pathological images, gene expression profiles, and clinical data used in this study are all derived from real-world clinical data in the TCGA database, with the model training data consistent with the data characteristics of actual clinical scenarios, eliminating the need for additional collection of special data and laying a foundation for the subsequent translation of the model into a clinical decision support tool.

## 5. Conclusions

Most of the existing studies on radiosensitivity prediction rely on genomic features, while ignoring the tumor microenvironment information in pathological images, which affects the accuracy of the predictions. Therefore, we proposed the multimodal deep learning model Resfusion. The model integrates pathological images, radiosensitive genomic features and clinical report features through the self-attention fusion module.

Based on the TCGA database, we constructed two datasets to predict tumor radiosensitivity in cancer patients: HNSC (200 cases) and BRCA (282 cases). These datasets were used to train and evaluate the proposed Resfusion model. Results from five-fold cross-validation demonstrated that Resfusion consistently outperformed existing multimodal survival prediction models on both datasets. However, this study did not incorporate radiomic features (e.g., CT or MRI) that are critical for radiotherapy planning [31,32,33], nor did it integrate fine-grained molecular characteristics such as immunohistochemistry or metabolomic profiles [34,35]. The absence of these complementary modalities limits the model’s ability to achieve a comprehensive and highly accurate prediction of tumor radiosensitivity. In future work, we plan to develop an extended multimodal framework that integrates radiomics with histopathology, genomics, and clinical information [36,37,38]. We also intend to collect multicenter clinical datasets covering multiple cancer types to further enhance the model’s generalizability and predictive robustness. Ultimately, our goal is to provide early and reliable predictions of radiosensitivity to support personalized and precise radiotherapy planning for cancer patients.

## Figures and Tables

**Table 1:** The details of used datasets.

Type	HNSC		BRCA	
	Positive	Negative	Positive	Negative
Train	120	41	191	35
Test	29	10	48	8

**Table 2:** Prediction results of various methods on HNSC dataset.

Model	Precision	Recall	F1	Accuracy	AUC
MMP	0.7582	0.7135	0.7358	0.6913	0.6795
Dyam	0.7038	0.6748	0.6889	0.6353	0.6568
Ours	0.8289	0.8057	0.8132	0.7300	0.7683

**Table 3:** Prediction results of various methods on BRCA dataset.

Model	Precision	Recall	F1	Accuracy	AUC
MMP	0.7438	0.7064	0.7251	0.7059	0.6874
Dyam	0.6935	0.6652	0.6793	0.6346	0.6531
Ours	0.8870	0.9164	0.8994	0.8339	0.7949

**Table 4:** Contribution of different data modalities to model performance on the HNSC dataset.

Type	Precision	Recall	F1	Accuracy	AUC
I	0.7686	0.8055	0.7866	0.6850	0.6606
G	0.8032	0.7179	0.7541	0.6550	0.6320
R	0.7716	0.7051	0.7348	0.6250	0.5762
I + R	0.7726	0.8013	0.7866	0.7000	0.6762
G + R	0.7672	0.7655	0.7636	0.6500	0.6651
I + G	0.7913	0.8124	0.7995	0.7000	0.7183
I + G + R	0.8289	0.8057	0.8132	0.7300	0.7683

**Table 5:** Contribution of different data modalities to model performance on the BRCA dataset.

Type	Precision	Recall	F1	Accuracy	AUC
I	0.8249	0.9130	0.8667	0.7868	0.6740
G	0.8013	0.8460	0.8230	0.7190	0.6408
R	0.8208	0.9048	0.8607	0.7766	0.6502
I + R	0.8689	0.9074	0.8994	0.8339	0.7165
G + R	0.8481	0.8661	0.8570	0.7661	0.6857
I + G	0.8517	0.8788	0.8641	0.7763	0.7305
I + G + R	0.8870	0.9164	0.9012	0.8339	0.7949

## Data Availability

The original contributions presented in the study are included in the article, further inquiries can be directed to the corresponding author.
